# An inpatient rehabilitation model of care targeting patients with cognitive impairment

**DOI:** 10.1186/1471-2318-12-21

**Published:** 2012-05-25

**Authors:** Katherine S McGilton, Aileen Davis, Nizar Mahomed, John Flannery, Susan Jaglal, Cheryl Cott, Gary Naglie, Elizabeth Rochon

**Affiliations:** 1 Department of Research, Toronto Rehabilitation Institute, E.W. Bickle Centre for Continuing Care, 130 Dunn Avenue, Toronto, ON, M6K 2R7, Canada; 2 L. Bloomberg Faculty of Nursing, University of Toronto, 155 College Street, Toronto, ON, M5T 1P8, Canada; 3 Toronto Western Research Institute, 399 Bathurst Street, Toronto, ON, M5T 2S8, Canada; 4 MSK Rehabilitation Program, Toronto Rehabilitation Institute, Hillcrest Centre, 47 Austin Terrace, Toronto, ON, M5R 1Y8, Canada; 5 Department of Physical Therapy, University of Toronto, 160-500 University Avenue, Toronto, ON, M5G 1V7, Canada; 6 Department of Medicine, Baycrest Geriatric Health Care Centre, 3560 Bathurst Street, Toronto, ON, M6A 2E1, Canada; 7 Department of Speech-Language Pathology, University of Toronto, 160-500 University Avenue, Toronto, ON, M5G 1V7, Canada

**Keywords:** Hip fractures, Dementia, Delirium, Cognitive impairment, Rehabilitation, Models of care, Mobility outcomes, Evaluation, Controlled investigation

## Abstract

**Background:**

The course and outcomes of hip fracture patients are often complicated by the presence of dementia and delirium, referred to as cognitive impairment (CI), which limits access to in-patient rehabilitation. In response to this concern, members of our team developed and piloted an in-patient rehabilitation model of care (Patient-Centred Rehabilitation Model; PCRM) targeting patients with hip fracture and CI (PCRM-CI). We are now conducting a 3-year study comparing an inpatient rehabilitation model of care for community dwelling individuals with hip fracture and CI (PCRM-CI) to usual care to determine whether it results in improved mobility at the time of discharge from inpatient rehabilitation.

**Methods/Design:**

A non-equivalent pre-post design is being used to evaluate the PCRM-CI compared to usual care. All community dwelling (private home or retirement home) patients following a hip fracture are eligible to participate. Recruitment of both cohorts is taking place at two facilities. Target accrual is 70 hip fracture patients in the PCRM-CI cohort and 70 patients in the usual care cohort. We are also recruiting 70 health care providers (HCPs), who are being trained to implement the PCRM-CI, and their unit managers. Patient data are collected at baseline, discharge, and 6 months post-discharge from an inpatient rehabilitation program. Evaluations include mobility, physical function, and living arrangement. Additional outcome variables are being collected from medical records and from the patients *via* their proxies. Data on the prevalence and severity of dementia and delirium are being collected. Staff data are collected at baseline and one year after implementation of the model to determine change in staff knowledge and attitudes toward patients with hip fracture and CI. Bi-monthly semi-structured interviews with unit managers have been conducted to examine factors and barriers influencing the model implementation. Data collection began in 2009 and is expected to be completed in 2012. The control cohort of 70 patients has been recruited, and 45 patients have been accrued to the intervention group to date.

**Discussion:**

Evaluation of this model of care is timely given the increasing proportion of persons with cognitive impairment and hip fractures.

**Trial registration:**

The study is registered at http://clinicaltrials.gov, Identifier NCT01566136.

## Background

A hip fracture is often a catastrophic event that results in significant impairment in mobility and function, and excessive institutionalization [1]. An estimated 25% to 75% of patients who are independent before their fracture can neither walk independently nor achieve their previous level of independent living within 1 year following the fracture [2]. Projections suggest that by 2041 the incidence of hip fractures in Canada will increase nearly four-fold [3], with an annual increase in economic burden of $2.4 billion per year [4], which is similar to other countries [5].

The course of hip fracture patients is often complicated by the presence of dementia and/or delirium. Dementia refers to a global loss of cognitive and intellectual functioning that gradually interferes with social and occupational performance [6]. Delirium is characterized by an acute decline in attention and cognition [7] and is linked to increased post-operative morbidity post-hip fracture [8]. Delirium may take weeks to months to diminish [9]. Both conditions are characterized by the presence of cognitive impairment (CI) across a spectrum of severity [8]. Two-thirds of cases of delirium occur in patients with dementia [10,11]. Studies have shown that 31 to 65% of older orthopedic patients have dementia and/or delirium [12]. As a result of the demographic shift to an aging population, the prevalence of dementia and the incidence of delirium in hip fracture patients are expected to progressively increase over the next 25 years [13]. In hip fracture patients, CI and low pre-fracture functional status are the most important predictors of poor prognosis regarding walking ability and return to independent living. [14].

Despite emerging evidence that patients with dementia and delirium can improve their mobility and function in rehabilitation [15,16], current health care services for people with a hip fracture and CI are fragmented and limited such that few patients are able to access rehabilitation services [4,17,18]. The lack of rehabilitation for these patients has negative consequences for the patients and the health care system: specifically, 1) hip fracture patients with CI have worse long-term mobility and functional outcomes compared to those without CI; and 2) many patients with CI have extended stays in acute care, some for several weeks, instead of being cared for in a less costly inpatient rehabilitation setting [19]. For those hip fracture patients with CI who are transferred to inpatient rehabilitation settings, the professional health care providers (HCPs) lack the necessary skills and knowledge about cognitive and behavioural strategies to care for them [20,21].

Despite research findings on rehabilitation, dementia, and delirium care, there are no rehabilitation models of care that combine these elements into a comprehensive model. To address this limitation we developed a model of care for all patients with hip fractures, with specific components of the model targeted for those patients with CI, referred to as Patient-Centred Rehabilitation Model (PCRM-CI). Our model is the first to include the following 5 components: rehabilitation management; dementia management; delirium prevention and management; staff education and support; and family/significant other support and education. Rationale for the separate components of the model is derived from the literature (Table 1).

**Table 1:** Components of the Patient-Centred Rehabilitation Model Targeted for Patients with CI (PCRM-CI)

**Components**	**Provider (s)**	**Effects**
1. Rehabilitation Management	Nurse, Physician, PT, OT, SW, pharmacist	**↑** function (Hurito *et al.*, 2000)
- assessments of function and cognition		**↑** mobility (Adunsky *et al.*, 1999)
- patient goal setting		**↑** return to previous living
- interdisciplinary rehabilitation treatments		arrangement (Naglie et al., 2002)
- weekly meetings to discuss progress of patient		
- discharge planning from week one		
2. Dementia Management	Nurse, Physician, PT, OT	**↓** agitation (Kovach *et al.*, 2006)
- assessment (MMSE)		**↑** function (Kovach *et al.*, 2006)
- relate well		
- environmental manipulation		
- abilities focus		
- personhood		
3. Delirium Management	Nurse, Physician, PT, OT	**↓** complications (Fick *et al.*, 2007)
- assessment (CAM)		**↓** LOS (Millisen *et al.*, 2004)
- prevention		
- treatment		
4. Staff education and support	APN	
- 1 day workshop on dementia, delirium, depression, care approaches, strategies to minimize behavioural and cognitive symptoms.		**↑** attitude towards patients with dementia (Zimmerman *et al.*, 2006; McGilton *et al.*, 2007).
- 8 in-services on the following topics: sleep, falls prevention, team building, pain management, incontinence, bowel management, roles and responsibilities of rehabilitation team and team building.		**↑** knowledge and skill of care of elderly clients (Moniz-Cook *et al.*, 1998; Cohen-Mansfield *et al.*, 1997)
- Advanced practice nurse mentoring at the bedside		**↑** support and improvement in work environment and **↑** satisfaction caring for clients with dementia (Wells *et al.*, 2001)
5. Family/significant other education and support	Nurse, SW	**↓** nursing home placement (Mittleman *et al.*, 1996)

Note: *OT* occupational therapist; *PT* physiotherapist, *APN* Advanced practice nurse, *SW* social worker.

Care of persons with dementia and delirium was guided by the 4 principles underlying the REAP (Relate well, Environmental manipulation, Abilities focused and Personhood) model of care conceptualized by the Principal Investigator (PI) [22]. First, staff’s ability to relate well is an essential component of HCP-patient interactions [23,24]. Depending on the patients’ level of CI, they may have difficulty understanding words or following directions [25]. Staff are taught techniques to relate effectively to their patients. Second, person-environment theory [26] argues for the need for synergy between person and environment. The environment must be modified and changed to accommodate the person’s changing needs and preferences. HCPs are taught to control the daily activity schedule of the patient in order to maintain a balance between high and low arousal states [27]. Third, abilities focused care [28] involves staff focusing on patients’ retained abilities. The ability of persons with dementia to perform Activities of Daily Living (ADLs) is influenced by their performance of purposeful movements, understanding of spatial orientation, and the ability to initiate activities. HCPs are taught to follow the steps of asking, cueing and demonstrating, before doing the activity for the patient. Fourth, Personhood [29] refers to knowing a person, becoming familiar with the individual, gaining knowledge of a person’s life, and identifying “what makes them tick” [30]. This process may involve partnering with families to gain needed knowledge. An understanding of the individual assists HCPs to become aware of the patient’s unmet needs, such as pain, often manifesting as behavioural agitation [31].

**Figure 1:**
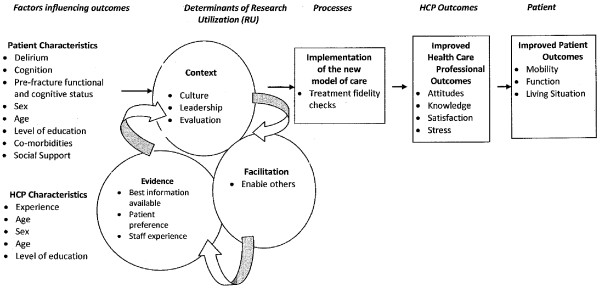
Patient-centred rehabilitation model of care targeting patients with CI (PCRM-CI).

The model was piloted on one musculoskeletal rehabilitation (MSK) unit at Toronto Rehabilitation Institute (Toronto Rehab) [32]. Results suggest that HCPs can learn to successfully rehabilitate hip fracture patients with and without CI utilizing this model [32]. Subsequently, the researchers decided to develop a demonstration project to address the following aims: 1) to evaluate the short and long-term patient outcomes associated with the PCRM-CI model of care as compared to usual care; 2) to determine if the PCRM-CI model can be replicated and sustained in a variety of community-based facilities that have different combinations of staff/patient ratios, staff skills, *etc.*; and 3) to establish, from a health human resource perspective, the influence on rehabilitation staff of caring for clients with CI. The overall purpose of this study is to establish a best practice model that will allow hip fracture patients with CI to regain their mobility and function. The findings of this research will have a major impact on the organization, management and delivery of services for the large and rising number of hip fracture patients with CI. The conceptual model for our study is depicted in Figure 1.

## Specific objectives

### Primary objective

To determine whether the PCRM-CI model, an inpatient rehabilitation model of care targeting community dwelling individuals with hip fracture and CI (dementia and/or delirium), results in improved mobility at the time of discharge from inpatient rehabilitation.

### Secondary objectives

1) To determine whether the PCRM-CI model for persons with hip fracture and CI results in: i) improved mobility at 6 months post-discharge from inpatient rehabilitation; ii) greater improvement in physical functioning at the time of discharge from inpatient rehabilitation and at 6 months post-discharge; iii) a higher proportion of patients returning to their previous living situation in the community at discharge and at 6 months post-discharge.

2) To determine whether the PCMR-CI model results in similar improvements in mobility at discharge from inpatient rehabilitation and 6 months post-discharge for patients with and without CI.

3) To evaluate whether the PCMR-CI model improves HCPs’ attitudes, knowledge, satisfaction, and stress, in caring for patients with CI.

4) To examine the processes by which the PCRM-CI model is implemented by describing: i) the evidence based components of the model that facilities adopt (*i.e.*, how rehabilitation staff in the facilities decide which components they will and can replicate); ii) the context (*i.e.*, evaluation of the preparedness of the context to embrace and sustain implementation); and iii) how the change is facilitated (*i.e.*, how and what supports the unit managers use to introduce, implement and sustain the new model) at the study sites.

## Methods/Design

A non-equivalent quasi-experimental before-after design is being used. Two sites have agreed to participate in the study. Multi-site sampling increases robustness by allowing for an assessment of comparisons and contrasts between the sites. Patient outcomes will be assessed at the time of discharge from inpatient rehabilitation and 6 months after discharge in cohorts of patients at both sites before and after implementation of the PCRM-CI model. The study has been registered at http://clinicaltrials.gov, Identifier NCT01566136.

### Setting and sample

The study is being conducted in two in-patient rehabilitation units at community rural sites. Site A is a 40-bed MSK rehabilitation unit in a 500-bed community hospital located 45 miles east of a large urban area. Site B is a 20-bed MSK rehabilitation unit at a small 120-bed community hospital. Ethics approval was obtained from both research sites and from Toronto Rehabilitation Institute.

The patient inclusion and exclusion criteria are: a) 65 years or older, b) admitted to rehabilitation directly from an acute care hospital after receiving surgery for a hip fracture; c) living in the community in a private home or residential setting (where an individual has services and supports but does not require 24 hour nursing care) prior to sustaining the hip fracture, d) able to speak and understand English; and e) have a family member or close friend who is familiar with the patient's pre-fracture condition and can act as a collateral informant. The patient exclusion criteria are: a) a pathologic hip fracture; b) a hip fracture associated with multiple trauma; and c) a previous hip fracture. All nursing staff, administrators and therapists caring for patients on the selected units will be invited to participate to assess their perceptions of the PCRM-CI. Written informed consent is obtained from both the patient and the caregiver/collateral informant at the first meeting following admission.

### Sources of data

Tables 2 and 3 list the quantitative and qualitative data we are collecting to meet the study objectives. In order to maximize the likelihood of observing a significant effect of the PCRM-CI model, we have selected outcome measures that have been shown to be sensitive to the model in our pilot work. We have focused our measurements at the point of discharge from rehabilitation and then at 6 months post-discharge, since recovery of ADLs often plateaus at this time [33].

**Table 2:** List of patient measures collected

**Concept**	**Construct**	**Standardized Measure where applicable**	**Time of data collection**		
			**Admission**	**Discharge**	**6 months Post-Discharge**
**Factors Influencing outcomes**					
Patient					
Characteristics	Age	NRS	x		
	Sex	NRS	x		
	Level of education	Interview	x		
	Co-morbidities	NRS	x		
	Cognition	MMSE	x	x	
	Delirium	Confusion Assessment Method;	x	x	
		if Yes, Delirium Index	x	x	
	Pre-fracture functional status	OARS	x		
	Pre-fracture cognitive decline	IQCODE	x		
	Social Support	Interview	x		
**Process Measures**					
Treatment Fidelity	Follow clinical pathway	PCRM-CI Checklist	Daily for First Week of Rehabilitation		
**Patient Outcome Measures**					
Patients’ outcomes	1) Mobility & Locomotion	FIM – Mobility subscale (5 items) (3 items) + Locomotion subscale (2 items)	x	x	x
	2) Motor Function	FIM – Motor subscale (NRS)	x	x	x
	3) Living Situation	Interview		x	x

**Table 3:** List of HCP measures collected

**Concept**	**Construct**	**Standardized Measurewhere applicable**	**Time of Data Collection**	
			**Pre-PCRM-CI Implementation**	**Post-PCRM-CI Implementation (one year later)**
**Factors influencing outcomes**				
HCP Characteristics	Age	Questionnaire	x	
	Sex	Questionnaire	x	
	Level of education	Questionnaire	x	
	Job category	Questionnaire	x	
	Years practiced	Questionnaire	x	
	Years worked in the facility	Questionnaire	x	
	Type of work settings before being employed in this facility	Questionnaire	x	
**Process Measures**				
Treatment fidelity	Participation in workshop and education	Education Log	The APN will track staff training	
**HCP Outcome Measures**	Attitudes towards patients with CI	ADQ	x	x
	Job satisfaction	Job Satisfaction Scale	x	x
	Work-related stress	WSI	x	x
	Knowledge of dementia care	Dementia Quiz	x	x
**Determinants of RU**				
HCPs	HCPs’ feedback on the implementation of the PCRM-CI	4 focus groups		x
Unit Managers	Unit manager’s feedback on the implementation of the PCRM-CI	Semi-structured interviews	Every 2 months for the period of the 1 year implementation of the model	

#### Quantitative data

##### Patient characteristics

Age, sex, education level, cognitive status (Mini-Mental State Exam; MMSE) [34], pre-fracture cognitive decline (Informant Questionnaire on Cognitive Decline in the Elderly; IQCODE) [35], delirium (Confusion Assessment Method; CAM) [36], delirium severity for those with delirium (Delirium Index) [37], pre-fracture functional status (Older Americans Resources and Services Instrument; OARS) [38], co-morbidities (the number of medical diagnoses), and social support (living alone or with someone). Level of education, level of social support, OARS, and IQCDODE, are being collected from the collateral informant.

In this way, patient’s recall bias will not be a concern. Sex, age, and co-morbidities will be obtained from the National Rehabilitation Services (NRS) database, as these data are collected by all rehabilitation facilities in Ontario. MMSE, CAM and Delirium Index are being collected by the research associate (RA) on admission and at discharge. Every week the RA will also do a CAM to screen for delirium for every patient enrolled in the study because up to 61% of elderly patients post-surgery may become delirious during their stay [7].

##### Patient outcomes

Mobility and physical functioning outcomes will be collected by the RA. Living situation will be collected from the collateral informant.

a) Mobility, the main outcome variable, will be measured by the Functional Independence Measure (FIM) locomotion and mobility subscales, referred to as the FIMM [39]. These subscales measure the ability to walk and climb stairs and the ability to transfer in and out of bed, toilet, and tub/shower. The FIMM has well established reliability and validity and has been shown to have a high sensitivity for detecting functional improvement in patients across a spectrum of functional abilities and comorbidities [32,39,40].

b) Physical Functioning will be assessed with the motor-FIM [41], which is the most widely used scale to assess the function of patients with CI following hip fractures [6,15,42-44]. The motor-FIM scale has a high sensitivity for detecting functional improvement in patients with varying functional status and varying degrees of comorbidities and has been closely associated with the amount of care required by CI patients [45]. FIM data have been validated for caregiver proxy report [43] and for telephone administration [46].

c) Return rates to previous living situation: The living situation will be classified into 4 categories: i) discharge to private community setting including home alone, home with spouse, home with other; ii) retirement home; iii) nursing home; and iv) acute care hospital, as categorized in previous studies with CI patients following rehabilitation [43].

#### Heath care practitioner

##### Demographic characteristics

The age, sex, education, experience, job category, and length of time working on the unit will be recorded in a questionnaire. We anticipate that most, if not all, staff will be willing to participate because of the endorsement of our research by administrators at the two institutions and based on previous HCP recruitment rates for evaluation studies [47,48].

#### HCP outcomes

a) Attitudes: The Approaches to Dementia Questionnaire (ADQ) will be used to assess HCPs’ attitudes. Two subscales indicate the staff member’s degree of hopefulness about dementia and the extent to which a person-centred approach is espoused [49]. The subscales have good reliability and have been validated against direct observation of the quality of staff care interactions with patients [50].

b) Knowledge of Dementia: The Dementia Quiz will be used to measure the knowledge that HCPs have about dementia [51]. The subscales have moderate reliability (.63 for knowledge and .57 for coping) and have demonstrated predictive validity for staff experienced with caring for persons with dementia.

c) Satisfaction Working with Patients with Dementia: This measure includes 21 items assessing satisfaction; each item is scored from 0 (not at all) to 4 (extremely) and summed to create a total score ranging from 0 to 84. Reliability and validity of the scale have been demonstrated [52].

d) Work Stress Inventory (WSI): We will use a modification of this measure that is derived by averaging the frequency of 45 stressors, each scored 1 (never-not at all) to 5 (often-very well). Reliability and validity of the scale have been demonstrated [52]. The subscales are grouped into three domains; task stressors, relationship stressors, and system stressors.

#### Qualitative data

##### Qualitative sampling and data collection

We will use theoretical (also referred to as purposive) sampling for our qualitative methods of data collection. Theoretical sampling is a specific type of non-probability sampling in which the objective of explanation determines the specific criteria by which a sample is selected. Qualitative data collection for Advanced Practice Nurse (APN), HCPs, and unit managers includes:

i. APN: The APN will assess treatment fidelity of staff by recording workshop participation, the extent to which sessions were conducted as intended, and barriers and facilitators regarding the delivery of the in-services (*e.g.* group dynamics, comprehension of material).

ii. HCPs: We will conduct focus group interviews with a sub-sample of the HCP participants of the study. The purpose of these focus groups will be to understand how and why the model of care worked or failed, the need for refinement, and the factors important for replication. Specifically, the evidence adopted, the context, and the facilitation methods that influenced the implementation will be sought [53].

iii. Unit manager: Following the introduction of the new model of care onto the unit, semi- structured interviews will be conducted with the unit managers of the two sites every 2 months over the 1 year period. The purpose of the interviews is to probe the evidence based components of the PCRM-CI model that the sites replicated (*i.e.*, how individuals at the sites decide which components they will and can replicate), the context, and how the change is facilitated.

## Intervention

### The patient centred rehabilitation model targeting patients with CI

Staff will be introduced to 5 components of the PCRM-CI in a one-day workshop prior to implementing the PCRM-CI model. They will then be provided with 8 additional educational sessions throughout the year. A manual detailing all aspects of training, including specifics on how to present the material, ideas for stimulating discussion, and case vignettes to illustrate training concepts was developed for our pilot study and is being used for this study.

### PCRM-CI components

1) Rehabilitation management post hip fracture includes conducting physical and cognitive assessments at admission (functional assessment, pain, depression, premorbid functioning assessment), deciding on person-centred goals with the patient and family, setting a discharge date early, weekly team meetings to discuss methods of improving rehabilitation for the patients, and intensive interdisciplinary daily rehabilitation [54,55]. Intensive daily rehabilitation includes hip range of motion, lower extremity strengthening, and daily increase of ambulation distance using the lowest level of assistance/aids required, with careful attention to pain management [56]. Additional rehabilitation management includes providing advice, training, encouragement, drug treatments, and help with equipment and daily living aids. The physiotherapist and occupational therapist provide therapy once a day for 1 hour, 5 days a week. In addition, nursing staff walk the patient to the bathroom and/or dining room several times a day. A clinical pathway that was used during Phase 1 has been given to staff for their use.

2) Dementia management involves assessment of cognition using the MMSE within the first 3 days of admission to rehabilitation, and use of the REAP model in practice. For example, when patients become anxious, staff are asked to focus on the REAP for assessment and intervention solutions. Do staff approach the patient and introduce themselves and their purpose? Does the environment need to be manipulated, (*e.g.* therapy at the bedside *versus* the gym) during the first week in order to make it more conducive for the patient who has CI?

3) Delirium management involves assessment for its presence using the Confusion Assessment Method (CAM) within 3 days of admission to rehabilitation, assessing severity with the Delirium Index if it is found to be present, addressing pre-disposing factors, and managing symptoms of delirium using the REAP model (*e.g.*, reorient patient, encourage family involvement, use clocks, normalize sleep) [7]. A delirium protocol developed in Phase I will be given to staff. It includes the CAM and interventions to manage delirium.

4) Staff education and support is an integral component of the model of care and as such an APN in gerontology will be hired for 1 year at the time that the model is implemented. As the dementia and delirium care components of the program are new for most staff, the APN will help HCPs learn how to conduct the assessments and interventions at the bedside. Furthermore, since many of the patients have multiple comorbidities and are frail, additional 30 minute educational sessions are provided to enhance HCPs’ knowledge base about non-pharmacological sleep interventions, falls assessment and prevention strategies, pain management, bladder re-training and bowel management. Three additional non-clinical in-services will focus on team building, nursing rehabilitation standards of practice, and roles and responsibilities of team members. An additional responsibility of the APN will be to “grow an expert” within the facility in order to sustain the new model of care beyond the period of the study.

5) Individualized family support and education will be provided by the team and will be reinforced on admission to the unit with a brochure which includes the goals of the rehabilitation program. Additional written aids will be given to family members including: information on delirium, resources available once the patient is home, and a discharge booklet to prepare for going home.

### Data collection

Ethics approval was obtained at the sites and the RAs (one at each site) have been trained in data collection procedures by the PI and a clinical psychologist for the cognitive scales (MMSE, CAM, Delirium Index, and IQCODE) and by a co-investigator for the FIM scale. The RAs have also been trained to conduct interviews, to take field notes and to record factors that may influence the adoption of the model into practice. Each RA is responsible for recruitment, data collection, and data management for one institution.

The study is running at the same time in both facilities. Staff have been asked to participate within the first few months of the project. Recruitment of patients in the usual care group has taken one year. Following this period, the workshop developed in phase 1 was given to all HCPs on the unit. The criteria for admissions were then revised to allow a larger number of community dwelling patients with CI to be admitted. Eight additional educational sessions, described earlier, have been given to the staff during a three-month learning period. Recruitment of patients for the PCRM-CI model of care has commenced, and accrual will take 8 months.

### Sample size

Sample size was determined based on the pilot data on two outcome measures and based on the requirement of the statistical methods that will be used to test the objectives. The primary outcome is the mobility gains score, the difference between mobility scores at discharge and at admission. In the Pilot Project at Toronto Rehab [32], the mean mobility gains score was 12.0 with a standard deviation of 5.7. This effect size was large: 2.1. The mean mobility gains score is expected to be higher for the PCRM-CI group than for the usual care group. If we assume that the mean mobility gains score for the usual care group will be 7.2 and the mean mobility gains score for the PCRM-CI group will be 12.1, with a common standard deviation of 5.7, the sample size estimate is 23 patients with an alpha of 0.05, power of 80% and a two sided *t*-test. In the Pilot Project, a secondary outcome was the rate of return to the community, which was 80% for patients with CI, and the PCRM-CI group is expected to maintain this rate. One can estimate that the rate of return to the community in the Usual Care group is about 50%. For comparison of these two proportions, with an alpha of 0.05 and power at 80%, the sample size estimate is 39 patients for each of the Usual Care and PCRM-CI groups. In order to allow for attrition, our goal will be to recruit additional patients beyond the minimums for a sample size of 140 patients (70 patients in each cohort).

Previous recruitment rates for evaluation studies have indicated that most staff are willing to participate. A sample size of 35 staff was estimated as sufficient to demonstrate changes in HCPs’ attitudes, based on results from the pilot project.

### Data analysis

Objective #1: The primary objective is to determine the impact of PCRM-CI on improvements in mobility (FIM locomotion and mobility) at discharge. At each site and for Usual Care and PCRM-CI groups, mobility is to be assessed at entry into rehab and at discharge from rehab. The change scores for patient outcomes will be compared with paired t-tests by cohort and site. In secondary analyses, multivariate regression will be used to examine the independent effects of each independent variable of interest on patient outcomes while controlling for other predictors and patient baseline factors**.**

Objective #2 (1 & 2): In the second instance, the mobility and physical functioning change scores and return to prior residence for Usual Care patients will be compared to those for PCRM-CI patients to assess the relative impact of the PCRM-CI model on outcomes for each site. Cross tabulations will be employed to assess rates of return to residence by rehab program and site. In particular, patients with CI are to be specifically targeted in the PCRM-CI model. We will compare means of mobility and physical functioning gains scores for patients with and without CI by program and site to assess the relative impact of the PCRM-CI model on patients with CI. In secondary analyses, multivariate regression will be used to examine the independent effects of each independent variable of interest on patient outcomes while controlling for other predictors and patient baseline factors**.**

Objective #2 (3): The introduction of the PCRM-CI model on the two units is meant to a) improve HCPs’ knowledge and attitudes toward working with hip fracture patients with CI, and b) measure the impact on provider satisfaction and work stress of working with these patients.

To meet this objective, descriptive statistics and paired t-tests analyses will be used for assessing the effects of the PCRM-CI model on provider experiences. Finally, the investigators will combine the qualitative assessments of HCPs in evaluating the impact of the PCRM-CI. Data pertaining to APN, HCPs and unit manager (interview and focus group transcripts, education log, field notes and treatment checklist) will be analyzed. Using an organizing/categorizing approach, each piece of qualitative data will be compared with every other piece of relevant data to develop insights into evidence, context and facilitation. The first step consists of reviewing all of the data sets and breaking the data down into discrete parts that are subsequently compared for similarities and differences (open-coding). The second step consists of putting the data together in new ways, by making connections between categories. The final step consists of systematically relating core categories to other categories, in order to integrate or refine analysis (selective coding). All the data sets will be reviewed to validate the core concepts [57].

## Discussion

In most rehabilitation facilities, CI makes patients ineligible for admission to rehabilitation. This leads to huge costs to the health care system as many of these patients await placement in acute care beds, become institutionalized, and within a year are immobile [15,19,58]. Based on results from our pilot work, we hypothesize that the PCRM-CI model can improve the mobility and functional outcomes of clients with CI, and allow them to return home at their prior level of function. This controlled study will provide evidence of the clinical effectiveness of this model. This demonstration project will also allow us to study the contextual factors important for understanding the transferability of the model into other jurisdictions, beyond academic teaching facilities. Study results will provide decision and policy makers with practical knowledge on models of care for persons with a hip fracture who have CI.

The study is critical given the increasing number of elderly persons with hip fractures, as it will contribute to meeting the emerging health needs of the population by improving the provision of services for elders with CI. Progress to date includes the collection of data on all 70 control subjects. At 6 months our sample was reduced by 8% due to attrition and mortality. Forty-five intervention subjects are enrolled with an end date of March 2012. From both facilities 65 health care professionals have agreed to participate in the study and have completed questionnaires at time 1 with the time 2 data to be completed at the end date.

Implementation of the model of care is timely, as the population is aging and the number of individuals with hip fracture and cognitive impairment is increasing. Sustainability after the life of the research project will be achieved by mentoring a staff person to be a ‘clinical coach’, *i.e.*, a future resource. If this rehabilitation model of care proves successful at the two study sites, there will be resources dedicated to implement the model of care into other rehabilitation facilities in its catchment area. This will likely also influence the practices of other hospitals, in keeping with their commitment to seniors’ care.

Given our extensive inclusion criteria and our partnering with community-based facilities, we anticipate that our study results can be generalized to hip fracture patients nationally and internationally. Hence, this study is timely and relevant to policy makers, health care administrators and providers, and the public.

## Competing interests

The authors declare that they have no competing interests.

## Authors’ contributions

KM wrote the first draft of this protocol, with AD, NM, JF, SG, CC, ER, GN contributing to the methods, discussion and editing. All authors have read and approved the final manuscript.

## Pre-publication history

The pre-publication history for this paper can be accessed here:

http://www.biomedcentral.com/1471-2318/12/21/prepub

## References

[B1] Naglie G Tansey C Kirkland JL Ogilvie-Harris DJ Detsky AS Etchells E Tomlinson G O’Rourke K Goldlist B Interdisciplinary inpatient care for elderly people with hip fracture: a randomized controlled trial CMAJ 2002 167 25 32 12137074 PMC116636

[B2] Magaziner J Hawkes W Hebel JR Zimmerman SI Fox KM Dolan M Felsenthal G Kenzora J Recovery from hip fracture in eight areas of function J Gerontol A Biol Sci Med Sci 2002 55 9 M498 M507 10995047 10.1093/gerona/55.9.m498

[B3] Papadimitropoulos EA Coyte PC Josse RG Greenwood CE Current and projected rates of hip fracture in Canada CMAJ 1997 157 1357 1363 9371065 PMC1228461

[B4] Wiktorowicz ME Goeree R Papaioannou A Adachi JD Papadimitropoulos E Economic implications of hip fracture: health service use, institutional care and cost in Canada Osteoporos Int 2001 12 4 271 278 10.1007/s001980170116 11420776

[B5] Baker NL Cook MN Arrighi HM Bullock R Hip fracture risk and subsequent mortality among Alzheimer's disease patients in the United Kingdom, 1988–2007 Age Ageing 2011 40 49 54 6 10.1093/ageing/afq146 21087990

[B6] Lieberman D Friger M Lieberman D Inpatient rehabilitation outcome after hip fracture surgery in elderly patients: a prospective cohort study of 956 patients Arch Phys Med Rehabil 2006 87 2 167 171 10.1016/j.apmr.2005.10.002 16442967

[B7] Inouye SK Delirium in older persons NEJM 2006 354 1157 1165 10.1056/NEJMra052321 16540616

[B8] Gruber-Baldini A Zimmerman S Morrison R Grattan L Hebel JR Dolan MM Hawkes W Magaziner J Cognitive impairment in hip fracture patients: timing of detection and longitudinal follow up J Am Geriatr Soc 2003 51 9 1227 1236 10.1046/j.1532-5415.2003.51406.x 12919234

[B9] Inouye SK Zhang Y Han L Leo-Summers L Jones R Marcantonio E Recoverable cognitive dysfunction at hospital admission in older persons during acute illness J Gen Intern Med 2006 21 12 1276 1281 10.1111/j.1525-1497.2006.00613.x 16965558 PMC1924736

[B10] Cole MG Delirium in elderly patients Am J Geriatr Psych 2004 12 1157 1165 14729554

[B11] Inouye SK Ferrucci L Elucidating the pathophysiology of delirium and the interrelationship of delirium and dementia J Gerontol A Biol Sci Med Sci 2006 61 12 1277 1290 10.1093/gerona/61.12.1277 17234820 PMC2645654

[B12] Magaziner J Simonsick EM Kashner TM Hebel JR Kenzora JE Predictors of functional recovery one year following hospital discharge for hip fracture: a prospective study J Gerontol 1990 45 3 M101 M107 2335719 10.1093/geronj/45.3.m101

[B13] Adunsky A Levy R Heim M Mizrahi E Arad M The unfavorable nature of preoperative delirium in elderly hip fractured patients Arch Gerontol Geriatr 2003 36 1 67 74 10.1016/S0167-4943(02)00058-4 12849100

[B14] Pioli G Davoli ML Pellicciotti F Pignedoli P Ferrari A Comprehensive Care Eur J Phys Rehabil Med 2011 47 2 265 279 21597436

[B15] Heruti R Lusky A Barell V Ohry A Adunsky A Cognitive status at admission: does it affect the rehabilitation outcome of elderly patients with hip fracture? Arch Phys Med Rehabil 1999 80 4 432 436 10.1016/S0003-9993(99)90281-2 10206606

[B16] Milisen K Foreman MD Abraham IL De Geest S Godderis J Vandermeulen E Fischler B Delooz HH Spiessens B Broos PLO A nurse-led interdisciplinary intervention program for delirium in elderly hip-fracture patients J Am Geriatr Soc 2001 49 5 523 532 10.1046/j.1532-5415.2001.49109.x 11380743

[B17] GTA Rehab Network Report 2004: Exploring the Hip Fracture and Joint Replacement Landscape in a Changing Context: Implication and Recommendations Toronto Retrieved from http://www.gtarehabnetwork.ca/uploads/File/reports/report-msk-march2006.pdf

[B18] Davis A Mahomed N Flannery J Brien H Saryeddine T Current status of musculoskeletal rehabilitation: an analysis of supply and provider viewpoints on future needs 2006 ACRUE, UHN, GTA Rehab Network Available at: http://www.gtarehabnetwork/publications.asp

[B19] Cott C Jaglal S Daniel I Drumm J MacKay C Markel F McKillop I Pink GH Soever L Hospital Report 2003: Rehabilitation. Joint Initiative of the Ontario Hospital Association and the Government of Ontario 2004 Hospital Report Research Collaborative, University of Toronto, Toronto

[B20] McGilton K Wells J Teare G Davis A Rochon E Calabrese S Naglie G Boscart V Rehabilitation of patients with dementia following a hip fracture; part 1: behavioral symptoms that influence care Top Geriatr Rehabil 2007 23 2 161 173

[B21] McGilton K Wells J Teare G Davis A Rochon E Calabrese S Naglie G Biscardi M Rehabilitation of patients with dementia following a hip fracture; part 2: cognitive symptoms that influence care Top Geriatr Rehabil 2007 23 2 174 182

[B22] McGilton K Lever J Mowat J Parnell L Perivolaris A Biscardi M Guideline recommendations to improve dementia care Alzh Care Quart 2007 8 2 109 115

[B23] Lawton MP Nahemov LE Ecology and the aging process 1973 American Psychological Association, Washington DC 619 674

[B24] McGilton KS Sidani S Boscart VM Guruge S Brown M The relationship between careproviders’ relational behaviors and residents mood and behavior in long-term care settings Aging Ment Health 2011 DOI:10/1080/13607863.628980 10.1080/13607863.2011.628980 22126318

[B25] Wells DL Dawson P A framework for developing nursing knowledge about the effects of dementia on older persons’ abilities J App Gerontol 2002 21 1 90 102 10.1177/0733464802021001006

[B26] Lawton MP Assessment, integration and environments for older people Gerontologist 1970 10 38 46 5463303 10.1093/geront/10.1_part_1.38

[B27] Kovach CR Taneli Y Dohearty P Schlidt AM Cashin S Silva-Smith MS Effect of the BASE intervention on agitation of people with dementia Gerontologist 2004 44 6 797 806 10.1093/geront/44.6.797 15611216

[B28] Dawson P Wells DL Kline K Enhancing the abilities of persons with Alzheimer’s and related dementias: a nursing perspective 1993 Springer Publishing Co, New York

[B29] Kitwood T The experience of dementia Aging Ment Health 1997 1 1 13 22 10.1080/13607869757344

[B30] Galik EM Resnick B Pretzer-Aboff I 'Knowing what makes them tick': motivating cognitively impaired older adults to participate in restorative care Int J Nurs Pract 2009 15 1 48 55 10.1111/j.1440-172X.2008.01721.x 19187169

[B31] Kovach CR Kelber ST Simpson M Wells T Behaviours of nursing home residents with dementia: examining nurse responses J Gerontol Nur 2006 32 6 13 21 10.3928/00989134-20060601-05 16773859

[B32] McGilton KS Calabrese S Davis A Mahomed N Flannery J Outcomes for older adults in an inpatient rehabilitation facility following hip fracture surgery Arch Gerontol Geriatr 2009 49 1 e23 e31 10.1016/j.archger.2008.07.012 18842307

[B33] Kagaya H Takahashi H Sugawara K Kuroda T Takahama M Quality of life assessment before and after lumbar disc surgery J Orthop Sci 2005 10 5 486 489 10.1007/s00776-005-0920-x 16193360

[B34] Cockrell JR Folstein MF Mini-Mental State Examination (MMSE) Psychopharm Bull 1988 24 4 689 692 3249771

[B35] Jorm AF A short form of the Informant Questionnaire on Cognitive Decline in the Elderly (IQCODE): development and cross-validation Psychol Med 1994 24 1 145 153 10.1017/S003329170002691X 8208879

[B36] Inouye SK van Dyck CH Alessi CA Balkin S Siegal A Horwitz RI Clarifying confusion: the confusion assessment method. A new method for detection of delirium Ann Intern Med 1990 113 12 941 948 2240918 10.7326/0003-4819-113-12-941

[B37] McCusker J Cole MG Dendukuri N Belzile E The delirium index, a measure of the severity of delirium: new findings on reliability, validity, and responsiveness J Am Geriatr Soc 2004 52 10 1744 1749 10.1111/j.1532-5415.2004.52471.x 15450055

[B38] Fillenbaum GG Smyer MA The development, validity and reliability of the OARS multidimensional functional assessment questionnaire J Gerontol 1981 36 4 428 434 7252074 10.1093/geronj/36.4.428

[B39] Siu AL Boockvar KS Penrod JD Morrison RS Halm EA Litke A Silberzweig SB Teresi J Ocepek-Welikson K Magaziner J Effect of inpatient quality of care on functional outcomes in patients with hip fracture Med Care 2006 44 9 862 869 10.1097/01.mlr.0000223738.34872.6a 16932138 PMC3033757

[B40] Eastwood EA Magaziner J Wang J Silberzweig SB Hannan EL Strauss E Siu AL Patients with hip fracture: subgroups and their outcomes J Am Geriatr Soc 2002 50 7 1240 1249 10.1046/j.1532-5415.2002.50311.x 12133019

[B41] Keith RA Granger CV Hamilton BB Sherwin FS The functional independence measure: a new tool for rehabilitation Adv Clin Rehabil 1987 1 6 18 3503663

[B42] Rolland Y Pillard F Lauwers-Cances V Busquère F Vellas B Lafont C Rehabilitation outcome of elderly patients with hip fracture and cognitive impairment Disabil Rehabil 2004 26 7 425 431 10.1080/09638280410001663148 15204479

[B43] Goldstein F Strasser DC Woodard J Roberts V Functional outcomes of cognitively impaired hip fracture patients on a geriatric rehabilitation unit J Am Geriatr Soc 1997 45 1 35 42 8994485 10.1111/j.1532-5415.1997.tb00975.x

[B44] Barnes C Conner D Legault L Reznickova N Harrison-Felix C Rehabilitation outcomes in cognitively impaired patients admitted to skilled nursing facilities from the community Arch Phys Med Rehabil 2004 85 10 1602 1607 10.1016/j.apmr.2004.02.025 15468018

[B45] Dodds T Martin D Stolov W Deyo R A validation of the functional independence measurement and its performance among rehabilitation inpatients Arch Phys Med Rehabil 1993 74 5 531 536 10.1016/0003-9993(93)90119-U 8489365

[B46] Segal ME Gillard M Schall R Telephone and in-person proxy agreement between stroke patients and caregivers for the functional independence measure Am J Phys Med Rehabil 1996 75 3 298 212 10.1097/00002060-199605000-00013 8663929

[B47] McGilton KS O’Brien-Pallas LL Darlington G Evans M Wynn F Pringle D The effects of a relationship-enhancing program of care on outcomes J Nurs Scholarsh 2003 35 2 151 156 10.1111/j.1547-5069.2003.00151.x 12854296

[B48] McGilton KS Irwin-Robinson H Boscart V Spanjevic L Communication enhancement: nurse and patient satisfaction outcomes in complex continuing care facility J Adv Nurs 2006 54 1 35 44 10.1111/j.1365-2648.2006.03787.x 16553689

[B49] Lintern T Woods B Phair L Before and after training: a case study of intervention J Dement Care 2000 8 1 15 17

[B50] Macdonald AJD Woods RT Attitudes to dementia and dementia care held by nursing staff in U.K. “non-EMI” care homes: what difference do they make? Int Psychogeriatr 2001 17 3 383 391 16252372 10.1017/s104161020500150x

[B51] Gilleard C Groom F A study of two dementia quizzes Brit J Clin Psych 1994 33 4 529 534 10.1111/j.2044-8260.1994.tb01149.x 7874044

[B52] Zimmerman S Williams CS Reed PS Boustani M Preisser JS Heck E Sloane PD Attitudes, stress, and satisfaction of staff who care for residents with dementia Gerontologist 2005 45 1 96 105 10.1093/geront/45.suppl_1.96 16230756

[B53] Kitson A Harvey G McCormack B Approaches to implementing research in practice Qual Health Care 1998 7 3 149 159 10.1136/qshc.7.3.149 10185141 PMC2483604

[B54] Beaupre LA Cinats JG Senthilselvan A Scharfenberger A Johnston W Does standardized rehabilitation and discharge planning improve functional recovery in elderly patients with hip fracture? Arch Phys Med Rehabil 2005 86 12 2231 2239 10.1016/j.apmr.2005.06.019 16344017

[B55] Jonsson A Gustafson Y Schroll M Hansen FR Saarela M Nygaard H Laake K Jonsson PV Valvanne J Dehlin O Geriatric rehabilitation as an integral part of geriatric medicine in the Nordic countries Dan Med Bull 2003 5 4 439 445 14694856

[B56] Cameron I Crotty M Currie C Finnegan T Gillespie L Gillespie W Handoll H Kurrie S Madhok R Murray G Quinn K Torgerson D Geriatric rehabilitation following fractures in older people: a systematic review Health Technol Assess 2000 4 2 1 111 i-iv 10702905

[B57] Strauss A Corbin J Basics of qualitative research: Techniques and procedures for developing grounded theory 1998 2 Sage, London

[B58] Jiang HX Majumdar SR Dick DA Moreau M Raso J Otto DD Johnston DWC Development and initial validation of a risk score for predicting in-hospital and 1-year mortality for patients with hip fractures J Bone Mineral Research 2005 20 3 494 500 10.1359/JBMR.041133 15746995

